# Selected Biochemical Blood Parameters and a Risk of Pressure Ulcers in Patients Receiving Treatment in Intensive Care Units

**DOI:** 10.3390/medicina57020177

**Published:** 2021-02-19

**Authors:** Dariusz Bazaliński, Beata Midura, Anna Wójcik, Paweł Więch

**Affiliations:** 1 Father B. Markiewicz Podkarpackie Specialist Oncology Centre, Specialist Hospital in Brzozów, 36-200 Brzozów, Poland; darek.bazalinski@wp.pl (D.B.); wojcik95@poczta.fm (A.W.); 2 Clinical Provincial Hospital No. 2 in Rzeszów, Rzeszów, 35-959 Rzeszów, Poland; midurowie@post.pl; 3 Institute of Health Sciences, College of Medical Sciences of the University of Rzeszów, University of Rzeszów, 35-959 Rzeszów, Poland

**Keywords:** undernutrition, albumins, pressure ulcers, intensive care

## Abstract

*Background and Objectives*: This study aimed to assess the level of selected biochemical parameters in venous blood and their potential effects on the development of pressure ulcers in patients treated in intensive care settings. *Materials and Methods***:** Fifty patients hospitalised in an intensive care unit (ICU) were enrolled for the study. The methods used included controlled observation, literature review and medical record analysis. The observation protocol applied in the study consisted of two parts comprising the basic information, sociodemographic data, results of laboratory tests (CRP, PCT, albumin, protein and haemoglobin concentrations) as well as the Braden Scale for Predicting Pressure Ulcer Risk. *Results***:** The subjects presented moderate to high risk of pressure ulcers, reflected by the mean score of 8.18 ± 1.3 points, with minimum and maximum scores of 6 and 12 points, respectively. Normal albumin level was identified in only five subjects (10.0%) while 45 subjects (90.0%) were found with results below the norm. A statistical relationship was observed between such variables as albumin concentration (*p* < 0.01) and total protein level (*p* = 0.007). The findings show a strong correlation between the score in the Braden Scale and the level of albumins (R = 0.55). *Conclusions***:** In our study, lower concentrations of albumins and total proteins correspond to a greater risk of pressure ulcers.

## 1. Introduction

Patients hospitalised at intensive care units (ICU) are particularly at risk of adverse health-related consequences and systemic complications [1]. A serious condition in patients is typically a result of multiorgan failure and immune response to metabolic stress. Treatments based on advanced invasive methods, such as mechanical ventilation, renal replacement therapy or vasopressors, despite their clinical importance, should not replace activities aimed at reducing the risk of malnutrition and the related consequences [2]. The stress of critical illness is associated with three different metabolic phases: acute, hypermetabolic and regenerative. Clinical consequences of metabolic response in the acute phase include loss of muscle mass and the resultant sarcopenia and hyperglycaemia [3]. Nonintentional loss of body mass resulting from metabolic response is a major risk factor for malnutrition and the subsequent onset of pressure ulcers. Rapid loss of fat-free body mass is a combined effect of the patient’s condition and the therapeutic activities related to procedural sedation and analgesia. Energy expenditure results from systemic inflammatory response syndrome (SIRS) associated with the injury and the possible infection as well as the surgical interventions and treatments administered [2].

There is a direct relationship between nutritional status and the development of pressure ulcers. Prevention of tissue damage caused by simple pressure and a lack of movement involves systematic, multistage activities performed mainly by nursing personnel. Simple procedures designed to relieve the pressure, to change the position of the body and to keep the skin clean in practice are carried out on a daily basis. The process of maintaining adequate nutritional status through personalised selection of dietary energy supply as well as by the administration of enteral/parenteral feeding is a much more advanced model, requiring the cooperation of specialists in various disciplines [4]. 

Basic laboratory tests to assess nutritional status include serum levels of albumin, prealbumin, transferrin, absolute lymphocyte count (ALC), C-reactive protein (CRP) and the retinol binding protein (RBP). Current research indicates that opinions regarding their usefulness vary. The main controversy relates to the numerous factors disturbing the interpretation process, such as the coexisting inflammatory process [5]. According to the guidelines developed by the expert group, EPUAP/NPIAP 2019, the assessment of malnutrition based on carrier proteins may be imprecise, especially in the acute phase of inflammation. Acute phase protein changes are not consistent or predictable in relation to weight loss and caloric restriction or nitrogen balance. This is reflective of the severity of the inflammatory response, as opposed to nutritional status. Marked inflammation increases the risk of malnutrition by increasing or altering metabolism and protein utilisation [6].

Monitoring and optimised nutritional management based on changes in body composition and biochemical parameters are among the many factors reducing the risk of pressure ulcers. By introducing reliable assessment of nutritional status (biochemical, anthropometric and clinical methods) as well as analysis of data from prophylactic treatments, it is possible to effectively prevent pressure ulcers in patients in the ICU [5,6]. Research has been undertaken to investigate the relationship between the biochemical parameters of peripheral blood and the risk of pressure ulcers in critically ill patients.

The present study was designed to assess selected biochemical parameters of venous blood and their potential effects reflected by the development of pressure ulcers in patients treated in an intensive care setting. 

## 2. Materials and Methods

### 2.1. Ethics

The study was conducted over a period of three months from September 2018 to January 2019. The study protocol was approved by the ethics committees of the involved institutions (Bioethics Commission at the University of Rzeszow: Decision no. 2018/01/07f on 7 January 2018) and performed according to the Declaration of Helsinki.

### 2.2. Setting and Participants

**Figure 1:**
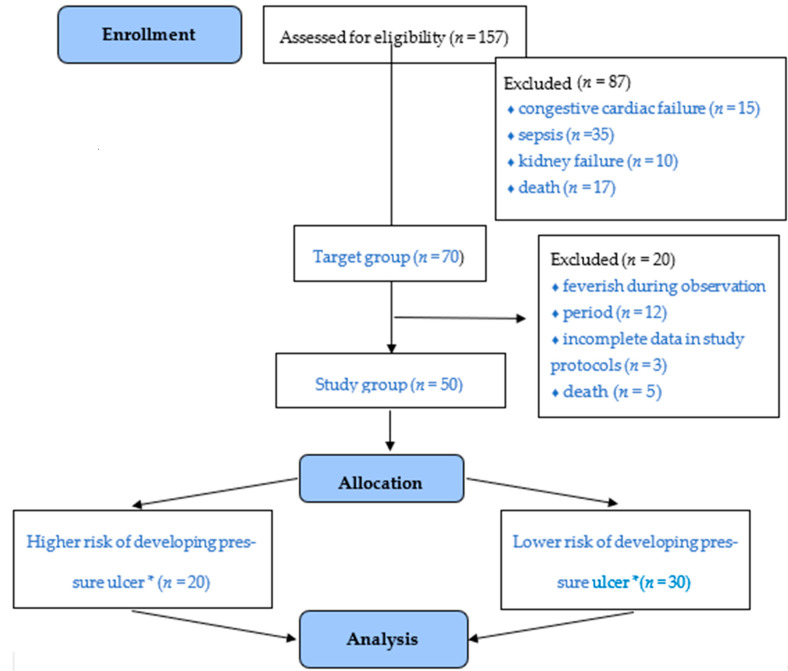
Flow chart demonstrating study participant selection. * The subjects were classified into two groups based on the score in the Braden Scale. Patients with a higher risk of developing pressure ulcers were included in the group with a score of ≤9 points, while those with a score over 9 points were placed in the lower risk group. This classification was created solely for the purposes of this study.

The authors would like to note that 157 people are hospitalised in this specific department every year. A total of 70 people were selected according to the adopted inclusion criteria. In terms of the study sample, the analysis involved 50 people—20 patients were excluded due to death (*n* = 5), being feverish (*n* = 12) during the observation period or finding incomplete history in the remaining 3 patients. Additionally, the subjects were divided according to the risk of developing a pressure ulcer (30 subjects with a higher risk vs. 20 subjects with a lower risk, according to the Braden Scale). Mean hospitalisation time was 10.86 days, with a 70% occupancy rate (the number of beds effectively occupied for curative care, divided by the number of available beds; Figure 1).

At the selection stage for determining the study sample, the following criteria were set to be met by the included group: patients who had stayed in the ICU for a maximum of 48 h, those with 4 points on the ECOG/WHO (Eastern Cooprerative Oncology Group) scale, those with a score of 10 or less on the GCS (Glasgow Coma Score), those with circulatory failure without congestive features, those with cancer and those 18 years of age or older. The set of criteria excluded those whose stay in the ICU exceeded 48 h, patients treated in other clinics, those with circulatory failure, those with kidney failure, those with sepsis and those below 18 years of age. 

Finally, fifty subjects qualified for the study were recruited among patients receiving treatment at the ICU of an intentionally selected hospital within the Podkarpackie Region. The study group comprised 18 women (36.0%) and 32 men (64.0%). The subjects ranged in age from 19 to 96 years, with the mean age amounting to 58.6 years ± 21.22 years. 

### 2.3. Study Design

The patient observation protocol developed for the needs of the study consisted of two parts. Part I comprised information related to sociodemographic data, health status and medical history, as well as GCS assessment of the level of consciousness, and assessment based on the Braden Scale for Predicting Pressure Ulcer Risk. The Braden Scale is a validated tool enabling assessment of pressure ulcer risk in ICU patients; it consists of six subscales assessing mobility, sensory perception, skin moisture, activity, friction and shear as well as nutritional status. Possible scores in the subscales range from 1 to 3 or 1 to 4; hence, the total score on the Braden Scale is in the range of 6–23; the lower results correspond to greater risk of pressure ulcers. Based on the total score, the risk is classified as very high (<9), high (10–12), moderate (13–14), low (15–18) or minimal (19–23). The authors applied a cut-off point of <9 to identify patients significantly at risk of pressure ulcers [7,8].

Part II of the protocol contained information related to laboratory tests: albumin and total protein levels in blood serum, CRP (C-reactive protein), PCT (procalcitonin), HGB (haemoglobin), WBC (white blood cells). Nutritional status was assessed based on albumin level (<3.5 g/dL) shown in the patient’s record, qualifying for nutritional therapy. The patients were assessed for pressure ulcer risk with the Braden Scale [5,9]. A total of 10 patients were assessed in the pilot study, and there were no inconsistencies found in the developed research tool. No changes were introduced in the developed tool after the pilot study.

### 2.4. Statistical Analysis

Statistical analysis was performed using Statistica 13.1 software (StatSoft, Inc., Tulsa, OK, USA). In terms of descriptive analyses, categorical variables were described as numbers and percentages. Data were reported as mean with a 95% confidence interval (CI) for quantitative measures, and as percentages for all categorical variables. Distribution normality was assessed using the Shapiro–Wilk test. The discrepancies in albumin levels in the four groups were assessed by the one-way ANOVA test, and the relationships between the laboratory test results and the Braden Scale were assessed according to the Spearman’s rank correlation test. A *p*-value below 0.05 was considered to be statistically significant. The statistical power of our study was 0.88, which is over the lowest recommended power of 0.88.

## 3. Results

Most subjects were hospitalised in the ICU due to conditions resulting from extensive neurosurgical procedures within the skull (42%), injuries of multiple organs (22%), conditions following sudden cardiac arrest (SCA) (14%), as well as inefficiency of organs, including respiratory failure due to pneumonia (24%). The comorbidities occurring in the study group, according to the medical records, included: diabetes (36%), hypotension (28.0%), hypertension (46%), atherosclerosis (58%), alcohol dependence (18%) and other conditions (e.g., multiple sclerosis, myasthenia, depression, chronic obstructive pulmonary disease (COPD) and multiple-organ failure) (28%). Detailed information is shown in Table 1.

The value of acute-phase protein (CRP reference range of 0.0–5.0 mg/L) at the time of the patients’ admission to ICU on average amounted to 129.56 mg/L ± 100.18 mg/L. Quality assessment of CRP showed normal values in four subjects (8.0%) and rates significantly above the normal range in 46 patients (92.0%). The value of procalcitonin (PCT reference range of 0.00–0.50 ng/mL) in the relevant group of patients on average amounted to 5.26 ng/mL ± 14.3 ng/mL and ranged from 0.04 ng/mL to 84.63 ng/mL. As for the quality of PCT, normal results were identified in 23 subjects (46.0%) while 27 subjects (54.0%) were found with values outside the normal range. The mean value of haemoglobin (HGB reference range of 13–17.5 L g/dL) in the study group amounted to 10.75 g/dL ± 2.07 g/dL. The test results showed normal haemoglobin levels in eight subjects (16.0%) while the results of 42 subjects (84.0%) showed abnormal values, ranging from 7.10 g/dL to 12.6 g/dL; these were patients with injuries. The mean level of white blood cells (WBC reference range of 4–11 103/with the µL) amounted to 11.64 103/µL ± 4.52 103/µL g/dL. 

Table 2 compares the above-described laboratory parameters in the group of subjects with a lower and higher risk of developing pressure ulcers. Statistical differences were noted in the case of albumin concentrations. In the lower-risk group, the albumin level was significantly higher (2.96 ± 0.49 vs. 2.48 ± 0.6; *p* = 0.004). 

Based on the albumin levels upon the patients’ admission, normal nutritional status was identified in five subjects (10.0%), mild malnutrition was found in nine subjects (18.0%), while 19 subjects (38.0%) and 18 subjects (36.0%) presented moderate and severe malnutrition, respectively. The values of albumin concentrations and CRP in the three groups did not differ significantly (*p* = 0.413) The lowest level of albumins in the blood occurred in the patients who had the highest CRP. 

**Figure 2:**
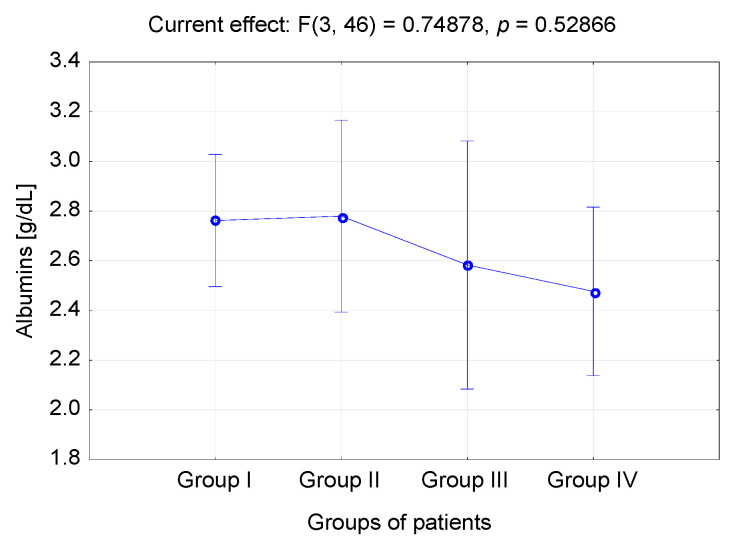
Risk of pressure ulcers among the subjects based on the Braden Scale. * (Group I—conditions following neurosurgical interventions and neurological disorders; Group II—injuries of multiple organs; Group III—condition due to SCA; Group IV—organ failure). F—result of F test (one-way analysis of variance (ANOVA)).

The average risk of pressure ulcers in the study population was assessed using the Braden Scale; all the subjects presented moderate to high risk of pressure ulcers. The scores on the Braden Scale were varied in the four groups (*p* = 0.039). Statistically significant differences were identified between the scores of Group II (injuries of multiple organs) and the results for patients in Group III (condition due to SCA) *p* = 0.038. The highest scores on the Braden Scale were found in Group II (81.84), and the lowest in Group III (78.86) (Table 3, Figure 2). 

The value of total protein amounted to 5.09 g/dL ± 0.98 g/dL. The findings show a relationship between the level of selected biochemical parameters and pressure ulcer risk. Statistically significant differences were found between such variables as albumin concentration (*p* < 0.001) and total protein level (*p* = 0.007) versus pressure ulcer risk. Detailed listing of the specific factors is shown in Table 4. 

**Figure 3:**
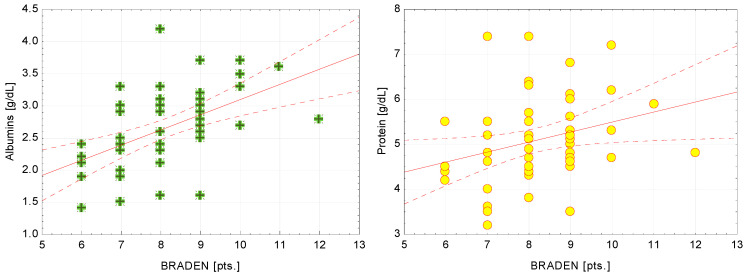
Effect of albumin and total protein levels on the risk of pressure ulcers, based on the Braden Scale.

The positive correlations suggested that lower risk of pressure ulcers (higher score on the Braden Scale) corresponded to higher albumin and total protein values. The correlation of the Braden score to albumin level was strong (R = 0.55) and to total protein level was moderate (R = 0.38) (Figure 3). 

## 4. Discussion

Malnutrition among hospitalised patients is still a common problem. It is estimated that 35–55% of patients admitted to hospitals are malnourished and approximately 20% of these require immediate nutritional therapy [4,10]. Monitoring of selected biochemical parameters as well as ongoing assessment of patients for a risk of pressure ulcers, with the use of recommended scales, contribute to decreased risk of pressure ulcers and complications [11]. By implementing personalised nutritional care, it is possible to prevent the adverse effects of malnutrition, while early diagnosis of the problem should be considered among priority tasks to be handled by medical personnel [2,3,4,5,6,7,12].

The performed tests showed normal albumin levels in 10% of patients, with simultaneous body weight loss. It is important to note that patients with the lowest albumin values also had the highest reported CRP values. This may suggest mobilisation of this protein type during the production of acute-phase proteins. Serum albumin is considered to be a hallmark of both inflammation and cachexia, or possibly malnutrition. However, because dietary restrictions do not always lead to serum albumin reduction, it has been suggested that the significance of inflammation outweighs the effects of malnutrition when measuring serum albumin levels [12].

Serra et al. assessed albumin levels in critically ill patients hospitalised in ICUs. They established that reduced concentrations correlated with incidence of presser ulcers. Protein supplementation and albumin level maintained at 2.8 g/dL or more favourably affected the process of pressure wound healing [13]. Similar observations were reported by Montalcini et al., who pointed out that low serum albumin, at a level < 3.1 g/dL, is a predictor for pressure ulcer onset and is associated with higher mortality. Signs of malnutrition identified based on this parameter were found in 21% of ICU patients [14]. Notably, serum albumin on average has a half-life of 12–21 days [15]. Therefore, assessment of nutritional status based on this parameter should be viewed in broad categories; according to Sobotka, low albumin concentration is an indicator of disease severity. Despite the low protein values exhibited by the majority of patients participating in the study, it is not recommended to use the total protein value for nutritional status assessment [16,17]. Researchers describe marasmus as a condition in which protein levels may be within the normal range while a loss of muscle mass, referred to as sarcopenia, is observed [5,12].

Opinions regarding the nutrition of critically ill ICU patients were presented by Fontes et al., who emphasised the difficulties related to the diagnosis of protein–energy malnutrition (marasmus). The authors carried out a study involving 185 critically ill patients in order to determine which method of assessing nutritional status is more effective. For this purpose, the researchers applied anthropometric and laboratory methods as well as a Subjective Global Assessment (SGA) scale. Based on the laboratory tests, protein–energy malnutrition was diagnosed in 100 (54%) patients, including 23 (12.4%) with severe malnutrition. The findings showed that malnourished patients were significantly more often readmitted to the ICU, and the mortality rate was 8.12 times higher compared to the patients with normal nutritional status. A comparison of the different methods for their consistency in diagnosing malnourishment showed their varied usefulness [18].

A study focusing on pressure ulcer risk assessment was also conducted by Terekeci et al., who investigated a group of 142 patients in ICUs. The researchers showed that the factors contributing to the incidence of pressure ulcers include age, low score on the Norton scale, duration of hospitalisation, low arterial blood pressure and hypoalbuminemia [19].

A study by Jaul et al. focusing on the development of pressure ulcers in patients with comorbidities showed that chronic conditions and other coexisting complicating factors associated with immobility, ischaemia and malnutrition contribute to the incidence of pressure ulcers, not only in hospital but also in the home setting and in care facilities [20]. The current study identified moderate to high risk of pressure ulcers in all the subjects, and no relationship was found between coexisting conditions and the risk of pressure ulcers. Ninbanphot et al. developed and validated a CAVE score (Cardiovascular-low Albumin-Ventilator-Edema) which, according to their evidence, is effective in predicting pressure ulcers in patients treated in intensive care settings. The authors applied the Braden Scale to evaluate the risk of pressure ulcers, and a four-stage scale to assess condition of the patients’ skin. They reported incidence of pressure ulcers in the ICU patients in the range of 9–11%. The instrument enabling evaluation of pressure ulcer risk takes into account the following factors: condition of the cardiovascular system, serum albumin level, application of mechanical ventilation and presence of oedema. The authors suggest that assessments based on the CAVE scale could effectively be performed by nursing personnel in clinical practice at ICUs [21]. Higher albumin levels corresponded to higher ratings achieved on the Braden Scale. There is no perfect method of assessing pressure ulcer risk; however, evaluation based on the Braden Scale takes into account patients’ nutritional status; the tool has been validated and it is preferred in assessing patients in the ICU [19,20,21,22,23].

Cramer et al., in their research related to methods of predicting the incidence of pressure ulcers at ICUs, applied electronic health records (EHR) to define patients’ profiles. The process took into account such data as Braden score, results of specific laboratory tests, diagnostic codes and the patients’ overall condition. The authors showed that initial EHR assessment could be an effective tool used in the screening of patients admitted to ICUs, allowing the identification of patients particularly at risk of pressure ulcers [24].

In their study focusing on the optimisation of nutritional care in the case of pressure injuries in hospitalised patients, Citty et al. report that more than one in two patients are affected by malnutrition, which contributes to the suppression of immune processes in the body, leading to the deterioration of the overall condition and promoting the development of pressure ulcers. Measures focusing on the prevention of pressure ulcers are necessary and multidimensional. The authors particularly emphasise the benefits of adequate nutritional care and report improved progress in pressure ulcer healing in patients subjected to such measures [25].

Intensive care is intended to create optimal chances for survival in critically ill patients who are in a potentially reversible, life-threatening condition. Management of critically ill patients requires highly specialised therapeutic and treatment procedures, including nutritional care. Traditionally, nutritional intervention in the case of critically ill patients involves the provision of nutrients in order to alleviate the effects of catabolic processes and to sustain fat-free body mass. Today, however, nutritional care is intended to reduce cell damage due to oxidative stress, and to beneficially modulate the immune response [26,27]. Preventing pressure ulcers in critically ill patients is extremely important. Assessment and provision of adequate nutrition, taking into account evidence-based guidelines (American Society for Parenteral and Enteral Nutrition—ASPEN/European Society for Clinical Nutrition and Metabolism—ESPEN), should be treated as an indispensable element of the medical care process.

Our study is obviously not free from some limitations. The presented results involve a small group of critically ill patients with additional burden of chronic disease, which may be reflected in the obtained study results and subsequent conclusions. Due to the fact that a complete blood cell count was not feasible, the study did not report total leukocyte count (TLC) values, which are the basic indicator in nutritional status assessment. Having considered the above, further studies would allow for a thorough analysis of the presented variables.

## 5. Conclusions

The study demonstrates that concentrations of albumins and total proteins correspond to a greater risk of pressure ulcers. The method based on the screening of patients with the Braden Scale and the monitoring of specific biochemical parameters can effectively be used to predict pressure ulcer risk in patients hospitalised in ICUs.

## Figures and Tables

**Table 1:** Baseline characteristics of patients.

Scheme		*n*	%
**Sex**	Women	18	36.0%
	Men	32	64.0%
**Reason for hospitalisation**	Conditions due to extensive neurosurgical procedures within the skull (G1)	21	42.0%
	Injuries of multiple organs (G2)	10	20.0%
	Conditions following sudden cardiac arrest (SCA) (G3)	7	14.0%
	Inefficiency of organs, incl. respiratory failure due to pneumonia (G4)	12	24.0%
**Comorbidities and/or past conditions**	Diabetes	18	36.0%
	Hypotension	14	28.0%
	Hypertension	23	46.0%
	Atherosclerosis	29	58.0%
	Alcohol dependence	9	18.0%
	Other	14	28.0%
**Age**	58.60 years ± SD 21.22 years (19–96)		
**Braden Scale**	8.18 points ± 1.3 points (6–12)		

**Table 2:** Comparison of the levels of laboratory parameters in the group with lower and higher risk of developing pressure ulcers according to the Braden Scale.

Parameter	Lower vs. Higher Risk of Developing Pressure Ulcers				
	Mean ± SD	Mean ± SD	Effect Size (95% Cl)	F (1. 48)	*p* Value
Albumins	2.96 ± 0.49	2.48 ± 0.6	0.88 (0.64–1.12)	**8.98**	0.004 *
CPR	104.05 ± 81.62	146.57 ± 108.83	0.45 (0.32–0.57)	2.21	0.143
PCT	2.51 ± 7.46	7.09 ± 17.33	0.37 (0.27–0.47)	1.24	0.271
Haemoglobin	10.46 ± 2.12	10.94 ± 2.05	0.23 (0.17–0.29)	0.64	0.428
WBC	12.6 ± 5.21	11.00 ± 3.96	0.35 (0.25–0.45)	1.52	0.223
Protein	5.37 ± 0.87	4.91 ± 1.01	0.49 (0.35–0.63)	2.81	0.100

* Bold characters indicate significant values (*p* < 0.05).

**Table 3:** Risk of pressure ulcers among the subjects based on the Braden Scale.

Albumins	Descriptive Statistics									
	*n*	$\bar{x}$	95.0% Cl	95.0% Cl	Me	Min.	Max.	Q1	Q3	SD
Group I	21	2.76	2.45	3.07	2.80	1.60	4.20	2.10	3.20	0.68
Group II	10	2.78	2.49	3.07	2.70	2.30	3.50	2.50	3.10	0.40
Group III	6	2.58	1.93	3.24	2.75	1.40	3.10	2.50	3.00	0.62
Group IV	13	2.48	2.12	2.83	2.40	1.50	3.70	2.20	2.90	0.59
*p*	F = 0.74 *p* = 0.528									

(Group I—conditions following neurosurgical interventions and neurological disorders; Group—injuries of multiple organs; Group III—condition due to SCA; Group IV—organ failure). F—result of F test (one-way analysis of variance (ANOVA)).

**Table 4:** Assessment of the relationship between selected biochemical parameters of venous blood upon admission to hospital and pressure ulcer risk, based on the Braden Scale.

Variables	R	*p* Value
Albumins and Braden	0.55	<0.001 *
CPR and Braden	−0.15	0.295
PCT and Braden	−0.18	0.219
Haemoglobin and Braden	0.00	0.986
WBC and Braden	0.16	0.282
Protein and Braden	0.38	0.007 *

R-value of Spearman’s rank correlation coefficient; * Bold characters indicate significant values (*p* < 0.05).

## Data Availability

The study did not report any data.
